# In Memoriam: Guy Dodson (1937–2012)

**DOI:** 10.3389/fendo.2013.00033

**Published:** 2013-03-21

**Authors:** Pierre De Meyts

**Affiliations:** ^1^Hagedorn Research Institute, Novo Nordisk A/SGentofte, Denmark

To the great sadness of his many friends, Guy Dodson passed away on Christmas eve 2012, 3 weeks before his 76th birthday. On January 10th, 2013, his latest joint publication appeared in Nature, describing crystal structures of insulin bound to several insulin receptor fragments containing one of the insulin binding sites, a major step forward in solving a riddle that had preoccupied him for over four decades (Menting et al., 2013). His co-authors dedicated the article to Guy, in recognition of his lifetime contribution to the study of the structure of insulin.

Guy was an outstanding X-ray crystallographer, internationally known for his highly influential work on insulin and on the mechanism of action of various enzymes, a.o. lipases and penicillin acylase.

**Figure 1:**
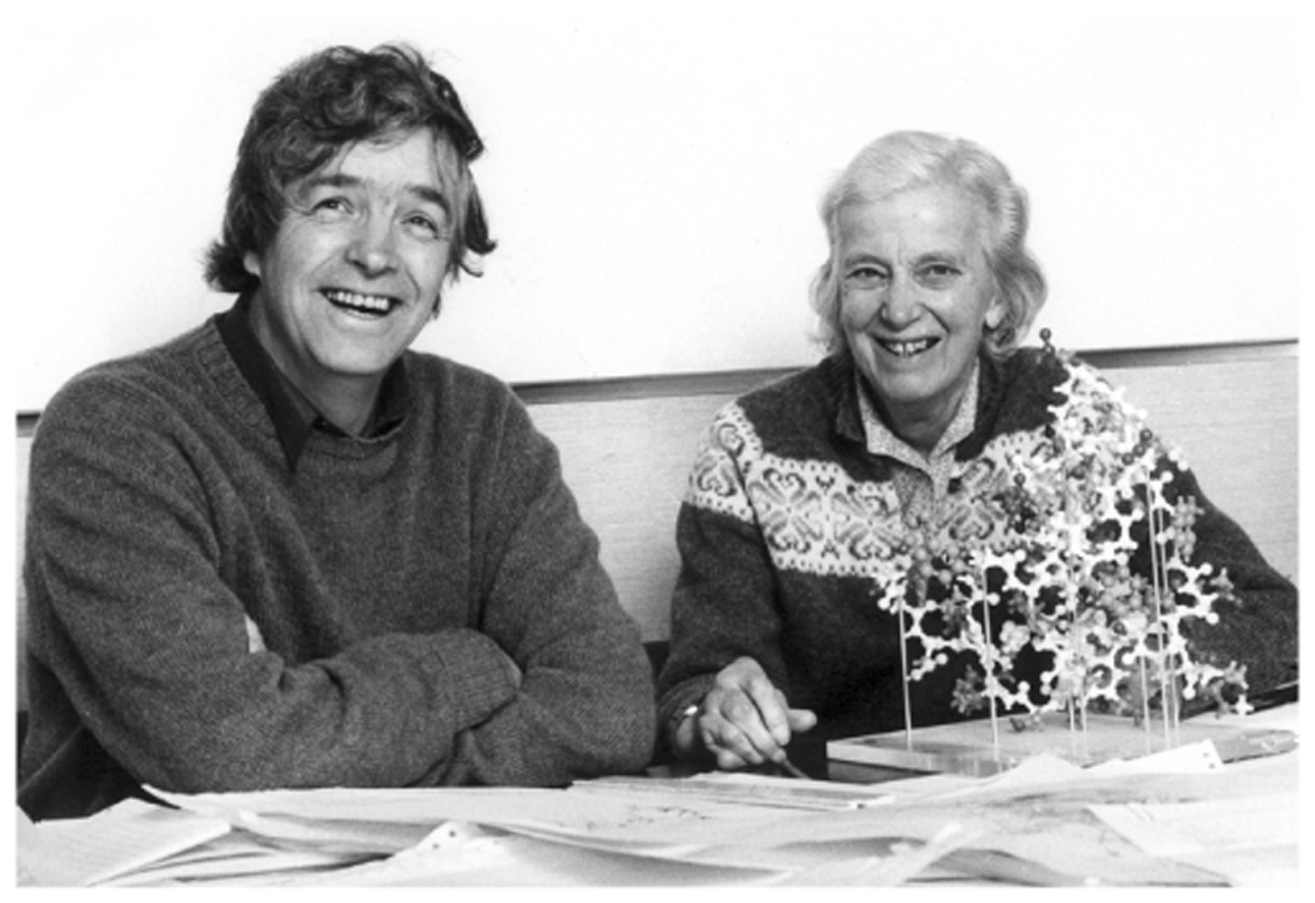
**Guy Dodson, Dorothy Hodgkin, and the insulin molecule**. Picture courtesy of Eleanor Dodson.

He and his twin brother Maurice were born on January 13th, 1937 in Palmerston North, New Zealand, from British immigrant parents. Guy did his B.Sc., M.Sc., and Ph.D. (in X-ray crystallography) at the University of New Zealand in Auckland. In 1962, he joined as a postdoc the laboratory of Dorothy Hodgkin in Oxford, 2 years before she won the Nobel Prize for solving the structure of penicillin and vitamin B12 (Figure 1). He stayed with her as a research fellow until her retirement in 1976. He married in 1965 his teammate Eleanor McPherson, an Australian with great mathematical skills. Together with two other fellows, Tom Blundell and M. Vijayan, they became the driving force that finally cracked the structure of insulin one summer day of 1969 – the month of the moon landing. Dorothy had been at it since 1935. Guy and his team continued to do seminal work on the structure and mode of self-assembly of insulin, with a strong emphasis on trying to understand how the structure relates to its biological mechanism of action. Among Guy's considerable output, two extensive articles from the Hodgkin lab became a “bible” for those of us working on insulin structure-activity: Blundell et al. (1972) and Baker et al. (1988). The later provides a painstaking description of the structural features of insulin at 1.5 Å resolution.

Guy and Eleanor moved to York University in 1976 where he became full professor in 1985; he became emeritus in 2004. In 1993 he was invited to establish a parallel X-ray crystallography effort at the National Institute of Medical Research in Mill Hill. There he focused on enzymes involved in malaria, proteins involved in tuberculosis and prions. He managed to keep both groups highly effective and productive. Guy became a Fellow of the Royal Society in 1994 and Eleanor in 2003.

The establishment of radioligand binding assays for studying the insulin receptor by Jesse Roth and colleagues at the NIH in 1971 transformed the way insulin structure-activity could be studied, limited up to then to mouse convulsion assays or tricky bioassays.

The use of synthetic, semisynthetic, and chemically modified insulins to explore the structure-function relationships of insulin was critical in the 1970s and early 1980s, when the advent of recombinant insulin vastly expanded the possibilities of making modified insulins, the structure of many of which was solved by Guy and his team. Guy also collaborated closely with the pharmaceutical industry in solving the structure of insulin analogs destined to clinical use.

These new methods helped the blossoming of an active international network with many fruitful collaborations, for which Guy continued to be a beacon. I feel very privileged to have been part of this community since my days at the NIH. I will mention Dietrich Brandenburg, Hans Gattner, Axel Wollmer, and the late Helmuth Zahn in Aachen; Tom Blundell's group after his move to Birkbeck College in 1976 and later to Cambridge; Jørgen Gliemann and Steen Gammeltoft in Copenhagen; Pierre Freychet and Emmanuel Van Obberghen in Nice; Don Steiner, Shu Jin Chan, and the late Howard Tager in Chicago; Jens Brange and Jan Markussen and their colleagues at Novo Nordisk in Denmark; Richard Jones and Peter Sönksen In London; Michael A. Weiss and Jonathan Whittaker in Cleveland; Panayotis Katsoyannis in New York; Stefan Emdin and Sture Falkmer in UmeÅ; the late Cecil Yip in Toronto; Ron Kahn, Steve Shoelson, and George King at the Joslin; the late René Humbel, Jürgen Zapf, and Rudy Froesch in Zurich (on the IGFs), Zhang You-shang, You-min Feng and colleagues in Shanghai; Colin Ward, Mike Lawrence and colleagues in Melbourne; Jiri Jiracek and Lenka Zakova in Prague and many more that I do not have room to include. Members of this network met on many memorable occasions. I fondly remember among others being with Guy at the European Workshop on insulin that I organized in 1978 in Brussels after my return from NIH, the Second International Insulin Symposium in Aachen in 1979 organized by Dietrich Brandenburg and Axel Wollmer, the International Symposium on Insulin and Insulin Action in Aachen in 1987, the conference organized by Guy in York in 1989 for the 20th anniversary of the insulin structure determination, the Alcuin Symposium in 2000 in Aachen for the retirement of Axel Wollmer, the symposium in York in 2004 for Guy's retirement and the 2005 symposium in Chicago for Don Steiner's 75th birthday.

Guy shared Dorothy's political commitments and was active in collaborations and contacts with scientists in China, India, and Cuba.

After retirement, Guy continued to be very active and I mentioned earlier the success of his and his associate Marek Brzozowski's collaboration with an international consortium led by Mike Lawrence in Melbourne and Mike Weiss in Cleveland, and other members of the abovementioned network, in solving a partial structure of the insulin receptor complex.

On a personal level, Guy was a very kind person of great charm, with no trace of arrogance, and with Eleanor created a very hospitable atmosphere in the lab. Guy's infectious and great enthusiasm for science was inspirational and is fondly remembered by all who worked with him.

Guy is survived by Eleanor, three sons, a daughter, and three grandchildren and his twin brother Maurice and his family.
